# Malignant Growths

**Published:** 1894-12-29

**Authors:** 


### MEDICAL PROGRESS AND HOSPITAL CLINICS.

[*The Editor will be glad to receive offers of co-operation and contributions from members of the profession. All letters should be addressed to* THE EDITOR, THE LODGE, PORCHESTER SQUARE, LONDON, W.]

### MALIGNANT GROWTHS.

The important question whether carcinoma is ever found as a primary disease in lymphatic glands, was discussed at the Pathological Society.[1] Mr. Bowlby had examined a case of squamous celled carcinoma of the glands of the groin, in which careful inspection of the pelvic tissue after death, and of the external soft parts, had failed to reveal the presence of any primary tumour. Mr. Butlin has referred to primary carcinoma of the lymphatic glands of the groin in chimney sweepers without discoverable primary tumour. Mr. Shattock has examined a case of squamous called carcinoma of the neck, but in that case could not discover any sign of its being in lymphatic glands, and considered the disease might have arisen in the residue of a branchial cleft.

One of the most important recent communications on the pathology of new growths is in a paper by Dr. Whitridge Williams, of Baltimore, on the "Histology and Histogenesis of Sarcoma of the Uterus."[2] This form of morbid growth occurs in two forms, either affecting the mucous membrane or the muscular wall of the uteus. Sarcoma of the endometrum is the most common, and may occur either as a diffuse infiltration of the mucosa, or as circumscribed growths which tend to assume a polypoid form. The growth may either remain limited to the mucosa, or may invade the muscular wall. It is very freely supplied with blood vessels and hæmorrhage often occurs, but sometimes large necrotic a eas arise. The polypoid form may easily be mistaken for an ordinary "fibroid polypus." Sarcoma of the parenchyma of the uterus is almost invariably circumscribed, and in naked eye appearance resembles ordinary fibroid growths. Like the myomata it may be subserous, interstitial, or submucous. Dr. Williams believes that cases described by Hutchinson, Callender, West, and Paget, as "recurrent fibroids of the uterus," were sarcomata. They may be composed of cells of any shape, giant cells even are sometimes found. One very interesting form occurs as grape-like bodies attached to the cervix. They look like cysts, and in a few cases cartilage and striated muscle fibres have been found in them. The great majority of such growths occur in patients under 20. They all died from regional matastases; true metastases, *i.e.*, growths in distant or ans only rarely occurred.

Such growths from the uterus as so-called carcino-sarcoma have been described by Virchow, Klebs, and others, but Whitridge Williams and other pathologists find it difficult to believe in such a combination with our present views as to the distinction between the two growths.

During the last few years another form of sarcoma of the uterus has been described. It occurs after the puerperium, and is formed of large cells, which closely resemble decidual cells, separated from one another by a fine reticulum. A typical case is related by Sänger. A young woman 23 years of age had an incomplete abortion in the eighth week. She bled for four weeks after it, but the uterus was cleaned out, and the hæmorrhage ceased, but she did not regain her health. The uterus enlarged, and a tumour appeared in the right iliac fossa. Great emaciation came on, and she died in seven months. Post mortem the uterus was found as large as if three or four months pregnant, and its walls occupied by masses of growth of a dark red colour and hæmorrhagic consistence. There were metastases of the same character in the lungs, diaphragm, iliac fossa, and tenth rib. Decidual-cell sarcoma, as this form of growth is called, is very rapidly fatal, and in every instance hæmorrhagic metastases were found in the lungs. Williams believes that some of the cases of so-called carcino-sarcoma belong to this variety of growth.

Whitridge Williams has collected the records of many cases described as the sarcomatous degeneration of a fibro-myoma of the uterus. Many have been described as such simply because they recurred, whereas perhaps other tumours grew in the same locality, and without any histological examination. Indeed, even growths histologically recognised as fibro-myomata have in some rare instances recurred, and even given rise to metastases. Such cases are recorded by Klebs,[3] Krische,[4] and Orth,[5] and in these cases there was no sarcomatous transformation. But Williams refers to many cases where a combination of sarcoma and fibro-myoma have been proved to exist in the same growth, and where the fibro-myoma is supposed to have developed into the more malignant type. And he considers that it is evident that fibro-myomata may be transformed into sarcomata either by the proliferation of the muscle cells themselves, or by the proliferation of the connective tissue cells between the muscle bundles. The form of growth in which the muscle cells themselves change into sarcoma he proposes to calls muscle-cell sarcoma.

One case of epithelioma of the clitoris[6] and two cases of epithelioma of the vagina have been recently recorded.[7] The epithelioma of the clitoris occurred in a woman only 29 years of age. In both the cases of epithelioma of the vagina, it was not the vaginal growth, but the gland enlargement in the groin which caused the patients to seek advice.

[1] Brit. Med. Journal, p. 966, Vol. I., 1894. [2] American Journal of Obstetrics, June, 1894, p. 721 [3] Meta-tasen von Myomen, Allg. Path., II., 704, 1889. [4] Ein. Fall von. Fibro myom, des uterus mit multiplen metastasen bei einer Geisteskranken. [5] Uterussarcom Lehrbuch der Spec. Path. Anat. Bd. ii. Lief. iii., 496. [6] Brit. Med. Journal, Vol. I., 1894, p. 1079. [7] Lancet, Vol. II., 1894, p. 257.

